# Therapeutic potential of adenovirus-mediated TFF2-CTP-Flag peptide for treatment of colorectal cancer

**DOI:** 10.1038/s41417-018-0036-z

**Published:** 2018-07-25

**Authors:** Zinaida A. Dubeykovskaya, Phaneendra Kumar Duddempudi, Huan Deng, Giovanni Valenti, Krystle L. Cuti, Karan Nagar, Yagnesh Tailor, Chandan Guha, Jan Kitajewski, Timothy C. Wang

**Affiliations:** 1 Division of Digestive and Liver Disease, Department of Medicine, Columbia University, College of Physicians and Surgeons, New York, NY USA; 2 Department of Biochemistry, Albert Einstein College of Medicine, 1300 Morris Park Avenue, Bronx, NY 10461-1602 USA; 3 Department of Radiation Oncology, Montefiore Medical Center, Bronx, NY USA; 4 Department of Physiology and Biophysics, College of Medicine, University of Illinois at Chicago, 835S. Wolcott Ave. E-202, Chicago, IL 60612 USA

**Keywords:** Genetic vectors, Colorectal cancer

## Abstract

TFF2 is a small, secreted protein with anti-inflammatory properties. We previously have shown that *TFF2* gene delivery via adenovirus (Ad-*Tff2)* suppresses colon tumor growth in colitis associated cancer. Therefore, systemic administration of TFF2 peptide could potentially provide a similar benefit. Because TFF2 shows a poor pharmacokinetic, we sought to modify the TFF2 peptide in a manner that would lower its clearance rate but retain bioactivity. Given the absence of a sequence-based prediction of TFF2 functionality, we chose to genetically fuse the C-terminus of TFF2 with the carboxyl-terminal peptide of human chorionic gonadotropin β subunit, and inserted into adenoviral vector that expresses Flag. The resulting Ad-*Tff2*-CTP-Flag construct translates into a TFF2 fused with two CTP and three Flag motifs. Administered Ad-*Tff2*-CTP-Flag decreased tumorigenesis and suppressed the expansion of myeloid cells in vivo. The fusion peptide TFF2-CTP-Flag delivered by adenovirus Ad-*Tff2*-CTP-Flag as well purified recombinant fusion TFF2-CTP-Flag was retained in the blood longer compared with wild-type TFF2 delivered by Ad-*Tff2* or recombinant TFF2. Consistently, purified recombinant fusion TFF2-CTP-Flag suppressed expansion of myeloid cells by down-regulating cyclin D1 mRNA in vitro. Here, we demonstrate for the very first time the retained bioactivity and possible pharmacokinetic advantages of TFF2 with a modified C-terminus.

## Introduction

TFF2 is abundantly expressed in the antrum of the stomach, but is also produced at a much lower level in immune cells. TFF2 belongs to the trefoil factor peptide family, which comprises a group of small, secreted proteins (TFF1, TFF2, and TFF3) with molecular masses of 7–12 kDa. Trefoil factor peptides display anti-inflammatory properties and play a significant role in mucosa protection and repair, presumably via receptors that remain to be fully characterized. However, the molecular basis for many of their diverse activities is largely unclear. We recently have shown that *TFF2* gene delivery via administration of an adenoviral construct (Ad-*Tff2)* greatly suppresses tumor growth in the azoxymethane/dextran sodium sulfate (AOM/DSS) colon cancer mouse model, predominantly by a mechanism that involves inhibition of the expansion of tumor-promoting myeloid-derived suppressive cells (MDSC) [1]. Transgenic overexpression of TFF2 or adenoviral delivery of TFF2 reduced myeloid proliferation and MDSC production, resulting in increased CD8+ T cells and decreased colon tumor growth.

Based on these observations, we believe that systemic administration of recombinant TFF2 could provide a substantial therapeutic benefit in the treatment of malignancies, particularly inflammation-associated cancers. However, pharmacokinetic studies have shown that intravenously administrated TFF2 is rapidly cleared from the circulation [2, 3]. To the best of our knowledge, there have been no studies in the past which have explored possible strategies designed to increase the in vivo half-life of trefoil peptides. Thus, in order to improve the pharmacokinetic profile of TFF2, we added the 28 amino acid carboxyl-terminal peptide (CTP) from the human chorionic gonadotropin β subunit to the C-terminus of the TFF2 coding sequence. The addition of CTP typically prolongs the half-life of peptides without affecting biological activity, secretion or receptor binding [4–6]. The CTP sequence contains four O-linked oligosaccharides sites that seem to prevent plasma clearance by increasing residence in the blood [7, 8]. However, such an approach is based on assumption that the carboxy-terminus of TFF2 is not critically important for its bioactivity. While large number of highly conserved amino acid residues have been identified in the primary sequence of trefoil peptides, the specific functional relevance of most of these sequences has not been assessed, due largely to the absence of appropriate bioassays. Thus, the absence of structural-based and sequence-based prediction of trefoil function has posed a significant hurdle to modifying TFF2 in order to prolong its half-life without affecting its bioactivity.

Despite these limitations, we used the adenoviral vector GV314 to create the adenovirus Ad-*Tff2*-CTP-Flag, which translates into the fusion protein, TFF2-CTP-Flag. Here, we show that, similar to Ad-*Tff2*, administration of Ad-*Tff2*-CTP-Flag decreases tumor number in AOM/DSS- treated mice and suppresses the expansion of CD11b^+^Gr-1^+^ cells (MDSCs) in the DSS-induced colitis model. After a single administration of Ad-*Tff2*-CTP-Flag, the fusion peptide TFF2-CTP-Flag was detected in the circulation for over one week longer compared with wild-type TFF2. Recombinant TFF2-CTP-Flag, when administered in vitro to splenic CD11b^+^Gr1^+^ cells, down-regulated cyclin D1 expression, although to a lesser extend compared with wild-type recombinant TFF2. Taken together, the results of this study indicate that TFF2 may be modified on C-terminus without eliminating its bioactivity. Recombinant C-terminus modified TFF2 circulates in the blood for a longer time compared with wild-type TFF2. Therefore, these findings provide a solid foundation to generate novel TFF2 peptides, based on modification of the TFF2 C-terminus.

## Materials and methods

### Mice

C57/BL6 wild-type mice were purchased from Jackson Laboratories (Bar Harbor, ME), *Tff2*-null and CD2-*Tff2* mice, both on C57BL/6 background, were previously generated in our laboratory and have been described earlier [1, 9]. All animal experiments were conducted in compliance with the National Institute of Health guidelines for animal research and approved by the Institutional Animal Care and Use Committee of the Columbia University. Mice were housed in a specific pathogen-free facility.

### Induction of colitis

In the experiments with DSS-induced colitis, 8–12-weeks old sex-matched *Tff2-null* and wild-type mice were given 2.5% DSS (m/w 36,000–50,000; MP Biomedicals, Solon, OH, USA) dissolved in drinking water provided ad libitum for 5 days, followed by plain water for next 14 days. Mice were analyzed at days 19.

### Tumor model

Mice (age 7–8 weeks) were injected intraperitoneally with AOM (10 mg kg^−1^ body weight) and after one week given 2.5% DSS water for 7 days. Four weeks later mice were treated with one dose 5 × 10^8^ pfu per mouse of Ad-*Tff2*-CTP-Flag (or Ad-*Tff2*) and Ad-Fc-CTP-Flag (or Ad-Fc) every two weeks via tail injection during 12 weeks and analyzed 4 weeks later after first injection. Macroscopically visible tumors were counted.

### Preparation adenovirus Ad-*Tff2*-CTP for fusion mouse TFF2-CTP-Flag expression

Adenovirus vector GV314 (pDC315-3FLAG-sv40-EGFP) was used in this study for preparation of a construct coding fusion TFF2-CTP-Flag. The full-length of mouse TFF2 open reading frame was inserted into the adenovirus vector GV314 by using BamHI/AgeI sites in frame fusion with 3× Flag, which was subsequently packaged into adenovirus. Construct pDC315-Fc-3FLAG-sv40-EGFP (Ad-Fc-CTP) coding Fc fragment of human IgG was used a as a negative control. Both vectors were purchased from Genechem Incorporation, (Shanghai, China). The recombinant adenoviruses were propagated with HEK293 cells and purified through two rounds of CsCl gradient ultracentrifugation and titrated by TCID50 method. Adenovirus was stored at −80 °C in storage buffer (10 mM Tris, 2 mM MgCl_2_, 10% glycerol, pH 7.5).

### Cloning, viral production, and purification of recombinant fusion TFF2-CTP-Flag protein

#### Cloning

Mouse TFF2-2xCTP-3xFlag DNA fragment was synthesized using Gblock (Integrated DNA Technologies) with Not1 and BamH1 cut-sites. This insert was cloned into Daedalus vector [10] using in-fusion cloning (Clontech). Lentivirus was generated on a larger scale using TPP tubes. Briefly, 2 × 10^7^ Freestyle 293-F cells in 10 ml of fresh Freestyle media (Invitrogen) were added to each TPP tube (Midsci). These cells were transfected with the DNA mix (8 μg of psPAX2, 4 μg of PMD2.G (Addgene) and 16 ug of the Daedalus vector) and 30 μl (at 1 μg/ml) PEI. 4-6 h post-transfection, 10 ml of fresh media was added to TPP tube. Then 24 h post-transfection, Valproic acid to a final concentration of 3 mM along with 10 ml of fresh media was added to TPP tube. The tubes were incubated in a Kuhner shaker, 37 °C, 8% CO_2_, 210 rpm for 72 h before harvesting the virus from the supernatant. This viral titer was calculated and stored at 4 °C until transduction step.

#### Viral production

1 × 10^7^ Freestyle 293-F cells, in 5 ml of fresh Freestyle media, were added to a 125 ml shake flask (Fisher Scientific), sufficient virus was then added for transduction at an MOI of ~10 as determined by the viral titer step, and the final volume was adjusted to 20 ml with growth media. The cells were incubated in Kuhner shaker incubator at, 37 °C, 8% CO_2_, 110 rpm for 4–6 h before the addition of 15 ml of fresh media. After 3 days of incubation in the shaker incubator, transduction efficiency was assayed by flow cytometry (using the GFP reporter). Cells showing ~99% transduction were scaled up to production volumes through rounds of dilution/expansion to necessary volume.

#### Purification

The supernatant was harvested by centrifugation at 500 × *g* for 15 min at 4 °C. The soup was passed through 0.45 μM filter to remove any remaining cells debris. Then the soup was concentrated to 1/10th of starting volume using 5000 Da cutoff vivaflow 50 cassettes (Sartorius). Then the concentrated soup was diluted to ten times using Equilibration buffer (20 mM Tris-HCl, 20 mM NaCl pH 8.0). Fusion TFF2-CTP-Flag protein from this diluted soup was purified using anion-exchange chromatography (Capto Q, GE life sciences). The bound protein was eluted using a salt gradient through elution buffer (20 mM Tris-HCl, 1 M NaCl pH 8.0). The eluate was then concentrated, filtered and applied to a size exclusion chromatography (HiLoad 26/600 Superdex 200 pg column, GE Healthcare, England) equilibrated with DPBS buffer. The fusion TFF2-CTP-Flag protein containing fractions were pooled, concentrated, filtered and checked for endotoxin levels (Kinetic-QCL^™^ Kinetic Chromogenic LAL Assay, Lonza).

### TFF2 administration and blood collection

Recombinant wild-type TFF2 was purified till homogeneity as described earlier [11] and given at a dose of 5 mg kg^−1^ of body weight (416 µM kg^−1^). The apparent molecular mass of recombinant fusion TFF2-CTP-Flag was around 38 kDa, due to a high level of glycosylation in HEK293 cells. Comparison of TFF2 and TFF2-CTP-Flag retention in the blood was based on equimolar concentration, and calculated from their apparent molecular mass as defined in western blot. Composite blood sampling was used for blood collection. Blood samples were collected at 0, 0.5, 1, 2, and 3 h time points after injection.

### In vitro test of recombinant fusion TFF2-CTP-flag produced by Freestyle 293-F cells versus TFF2

Comparison of the activity of recombinant fusion TFF2-CTP-Flag produced by Freestyle 293-F versus wild-type TFF2 was done in vitro test with CD11b^+^Gr1^+^ cells. *Tff2*-null mice were given 2.5% DSS water for consecutive 5 days, and then mice were given regular tap water for 14 days. On day 19 after start of treatment mice were sacrificed and CD11b^+^Gr-1^+^ were sorted from the spleen. CD11b^+^Gr-1^+^ cells (8 × 10^5^ per well) were cultured 7 days in DMEM media with 10% FBS and2.5 ng ml^−1^GM-CSF in the presence of wild-type TFF2 and fusion TFF2-CTP-Flag at the indicated concentrations, then total mRNA was isolated and cyclin D1 mRNA was measured.

### Western blot analysis

For western blot analysis, 5 μl of serum (or urine where indicated) were separated by using pre-cast 10–20% SDS-polyacrylamide gels (Bio-Rad) and transferred onto Immobilon-P PVDF membrane (0.22 µm, Millipore). All blots were blocked in 5% skim milk in Tris-buffered saline (TBS) plus Tween 20 (0.05%) for 1 h. Affinity-purified primary polyclonal rabbit antibodies were produced against the C-terminus of mouse TFF2 (New England Peptide) and used at concentration 1 µg ml^−1^. Anti-Flag antibody (Cell Signaling) was used at dilution 1:2000. Blots were developed with Pierce ECL Plus Western Blotting Substrate (Pierce ECL).

### RT-PCR

Liver samples were placed in RNAlater stabilization solution (Ambion) and kept at −80 °C until use. Total mRNA was isolated by using NucleoSpin RNA kit (Macherey-Nagel) according to the manufacturer’s instructions. RNA quantity was evaluated by using NanoDrop (Thermo Fisher Scientific). cDNA was synthesized from 1 μg total RNA in a volume of 20 μl by using random hexamer primers with the first-strand cDNA synthesis kit (Invitrogen, Carlsbad, USA). Amplifications were carried out with a total volume of 20 μl with TaqMan Universal PCR Master mix and following primers: TFF2 (Mm.PT.58.10220652 /56-FAM/AC TCT CAG T/Zen/A GTG ACA ATC TTC CAC AGA CT/3IABkFQ) and reference gene Hprt (Mm.RT.39a.22214828 /56-FAM/CT TGC TGG T/Zen/G AAA AGG ACC TCT CGA A/3IABkFQ).

Cell cycle related gene cyclin D1 was tested by using mouse primers for cyclin D1 (forward) 5′-GCGTACCCTGACACCAATCTCC-3′, (reverse) 5′-CCTCTTCGCACTTCTGCTCCTC-3′, reference mouse gene GAPDH (forward) (5′-ACGGACCCCAAAAGATGAAG-3′; (reverse) 5′-TTCTCCACAGCCACAATGAG-3′ and PowerUp™ SYBR Green Master Mix (Applied Biosystems). Assays were performed on ABI 7500 thermal cycler (Applied Biosystem). All determinations were performed in technical triplicate. The relative abundance of each mRNA was calculated by the ΔΔ*C*_T_ method normalizing to housekeeper gene expression with standardization to one reference sample.

### Statistical analysis

Graphs and analyses were calculated using Prism 6.0 for Mac OSX (GraphPad software, Inc) and Excel. Either a two-tailed Student’s *t*-test or one-way ANOVA was used to evaluate significance between two groups for parametric and non-parametric data, respectively. The following designations apply to all figures: *p < 0.05, **p < 0.01, ***p < 0.001. *p*-values < 0.05 were considered significant. Data were expressed as means ± SD of the means.

## Results

### Adenovirus-mediated delivery of fusion TFF2-CTP-Flag

**Fig. 1:**
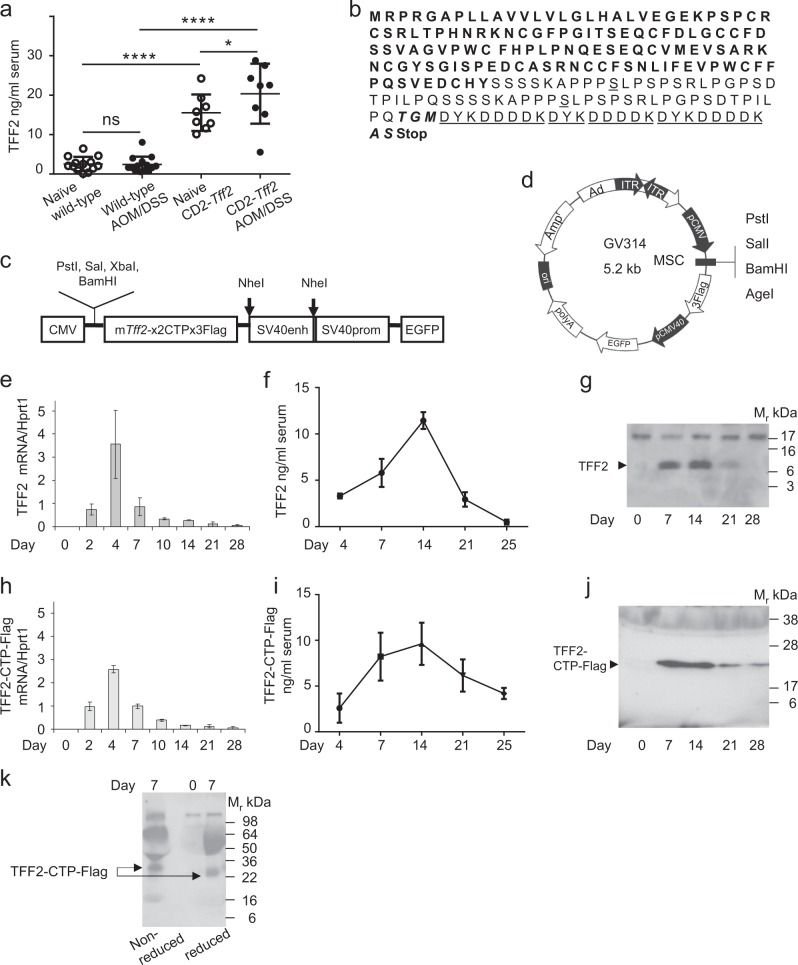
Identification of wild-type TFF2 and fusion TFF2-CTP-Flag protein delivered by Ad-*Tff2* and Ad-*Tff2*-CTP-Flag in the blood. **a** TFF2 level assayed by ELISA in the blood from naive wild-type and CD2-*Tff2* transgenic mice and mice at time point 6 months after induction of tumorigenesis with AOM/DSS treatment. Dunn’s multiple comparisons test after one-way ANOVA test, ns, non-significant, *p < 0.05, ****p < 0.001. **b** Amino acid sequence of fusion protein TFF2-CTP-Flag: TFF2 amino acid sequence—bold regular, CTP—regular, *S*—underlined, original cysteine substituted for serine, additional amino acids residues -bold italic shadow, Flag-underlined regular, Stop—stop codon. **c** Schematic presentation of fusion construct *Tff2*-2CTP-3Flag. **d** Map of GV314 vector with inserted TFF2 gene. **e**–**j** Time course of TFF2 and TFF2-CTP-Flag level after single administration of adenoviruses Ad-*Tff2* and *Tff2*-CTP-Flag. *Tff2*-null mice were injected with 5 × 10^8^ pfu of Ad-*Tff2* or Ad-*Tff2*-CTP-Flag and sacrificed at indicated times post infection. Time-course of *Tff2* mRNA (**e**) and *Tff2*-CTP-Flag mRNA (**h**) expression in the liver. Total mRNA was isolated from the liver and then *Tff2* mRNA was detected by qPCR, fold of change normalized on housekeeper *Hprt* mRNA. For each time point 3 mice were used, graphed as mean ± SD. **f**, ** i** Kinetic of TFF2 protein and TFF2-CTP-Flag fusion protein in the blood assessed by ELISA and expressed as mean ± SD at each time point, three animals per group. **g**, **j** western blot for TFF2 and TFF2-CTP-Flag in mouse serum. Five microliter of mouse serum were loaded in each lane and western blot was developed with antibody produced against C-end of TFF2 molecule, arrows indicate the position of TFF2 and TFF2-CTP-Flag with calculated sizes 12 and 25–26 kDa accordingly. **k** Western blot of blood sample (5 µl) from *Tff2*-null mouse taken on day 7 after Ad-*Tff2*-CTP-Flag administration. Western blot was developed with anti-Flag antibody; arrows indicate the positions of TFF2-CTP-Flag fusion under reduced and non-reduced conditions

We found that basal TFF2 levels in the serum of naive transgenic CD2*-Tff2* mice were in the range of at least one order of magnitude higher compared to naïve wild-type animals, and these levels of TFF2 were sustained after the treatment with AOM/DSS (Fig. 1a). Given that CD2-*Tff2* mice are more resistant to colon carcinogenesis, and treatment with adenovirus Ad-*Tff2* increases circulating levels of TFF2 and confers resistance to colon tumorigenesis, we assumed that the circulating level of TFF2 in CD2-*Tff2* mice was a prerequisite for the antitumor effect. Since systemically administered recombinant TFF2 is rapidly cleared from circulation, we added the nucleotide sequence coding for CTP to the carboxyl end of TFF2, and subcloned the resulting construct into the adenoviral vector GV314, which also expresses Flag and GFP markers (Fig. 1b–d). The final construct encoded for a full-length mouse TFF2 fused with two CTP and three Flag (tag) molecules, with a theoretical molecular mass 23 kDa for the whole fusion. The original cysteine in the CTP sequence was replaced with serine. As a negative control for many of the experiments, we used the original adenovirus vector Ad-Fc-CTP-Flag.

Since liver shows the highest level of transgene expression following infection with adenovirus [12], we first confirmed TFF2 mRNA expression in the liver of *Tff2*-null mice after a single dose (5 × 10^8^ pfu per mouse) of Ad*-Tff2*-CTP-Flag versus Ad-*Tff2*. In both cohorts of mice, similar kinetic profiles of TFF2 mRNA transcripts were observed in the liver, with maximum abundance at day 4 and a decline to basal levels by the end of third week (Fig. 1e, h). We assayed TFF2 peptide levels by ELISA and performed western blots with an antibody produced to the TFF2 C-terminus in order to detect the TFF2-CTP-Flag fusion. We found that concentrations of the native TFF2 peptide, as well the fusion TFF2-CTP-Flag peptide, reached the highest level at days 7–14 after a single injection of Ad-*Tff2* and Ad-*Tff2*-CTP-Flag (Fig. 1f, g, i, j). We identified the TFF2-CTP-Flag fusion peptide in the serum of *Tff2*-null mice by western blot as a specific band with molecular mass 25–26 kDa, which is close to the predicted size (Fig. 1j). The fusion TFF2-CTP-Flag was still clearly detectable at a day 25, in contrast to wild-type TFF2, which was barely detectable by western blot after 21 days. We also used a commercial anti-Flag antibody in western blot, and under reducing conditions we identified the fusion protein TFF2-CTP-Flag with a molecular mass of 25–26 kDa, similar that detected with an anti-TFF2 antibody under the same conditions (Fig. 1k). In parallel, an immunoreactive band with a slightly lower motility and higher molecular mass (~30 kDa) was detected with an anti-Flag antibody under non-reducing conditions.

### **In vivo** suppression of tumor growth and expansion of CD11b^+^Gr-1^+^ cells was not affected by fusion TFF2 with CTP-Flag

**Fig. 2:**
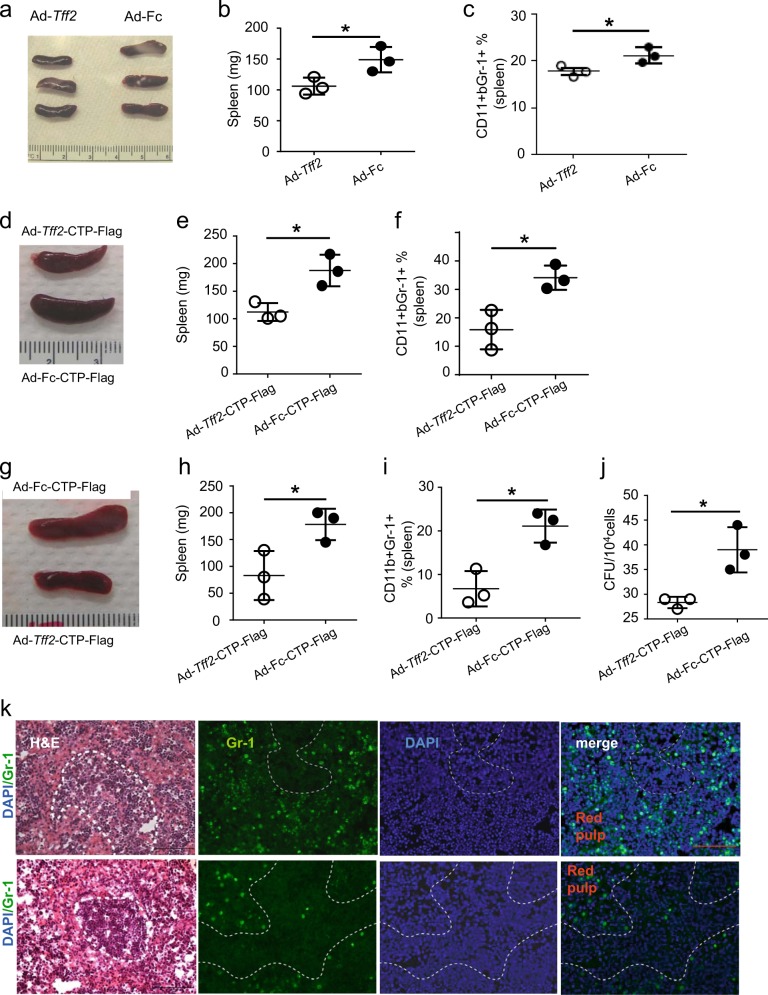
Ad-*Tff2* or Ad-*Tff2*-CTP-Flag reduces splenomegaly and proportion of myeloid cells in the spleen of wild-type and *Tff2*-null mice. **a**–**f** Wild-type mice were treated 2% DSS for 5 consecutive days, then on day 12 post treatment with DSS single injection of Ad-*Tff2* (**a**–**c**) or Ad-*Tff2*-CTP (**d**–**f**) and Ad-Fc or Ad-Fc-CTP-Flag as controls. Dose 5 × 10^8^ pfu was administered in tail vein. Mice were sacrificed on day 19. Spleen appearance (**a**, **d**), spleen size mass (**b**, **e**) and proportion of CD11b^+^Gr-1^+^ cells (**c, f**) in spleen, unpaired *t*-test, *p < 0.05. Two experiments with three mice in each group have been done. **g**–**i** Ad-*Tff2-*CTP-Flag reduces splenomegaly (**g**, **h**) and proportion of CD11b^+^Gr-1^+^ cells (**i**) in the spleen of *Tff2*-null mice, unpaired *t*-test, *p < 0.05. One experiment with three mice in each group has been done. **j** Administration of Ad-*Tff2*-CTP-Flag decreases granulocytes/macrophages colonies-forming units in the spleen of wild-type mice treated with DSS. Data obtained from mice treated with 2% DSS for 5 consecutive days and sacrificed on day 19, unpaired *t*-test, *p < 0.05. **k** Immunostaining for Gr-1 in the red pulp area in spleens of *Tff2*-null mice treated DSS water and received Ad-Fc-CTP (upper raw) or Ad-*Tff2*-CTP (lower raw), day 19. Bar size is 100 µm

Next we evaluated bioactivity of the modified TFF2 peptide by using DSS-induced colitis and AOM/DSS-induced colon cancer models. We have previously demonstrated in a model of DSS-induced colitis that, in contrast to CD2*-Tff2* mice, *Tff2*-null counterparts develop massive splenomegaly by 19–21 days due to the expansion of splenic myeloid CD11b^+^Gr-1^+^ cells. Based on this observation, we hypothesized that an injection of Ad-*Tff2* or Ad-*Tff2*-CTP-Flag would suppress the expansion of myeloid CD11b^+^Gr-1^+^ cells, leading to smaller spleen size. Therefore, we first induced colitis in wild-type mice (as well in *Tff2*-null mice) using 2.5% DSS in the drinking water for 5 days, followed on day 12 by the administration of Ad-*Tff2* or Ad-*Tff2*-CTP-Flag, with additional mice treated with Ad-Fc or Ad-Fc-Flag as controls. Mice were sacrificed on day 19 from start DSS treatment. At necropsy, we found that the spleens from the mice injected with Ad-*Tff2* or Ad-*Tff2*-CTP-Flag were smaller compare to those from the mice injected with Ad-Fc or Ad-Fc-CTP-Flag (Fig. 2a, b, d–e, g–h). Consistent with the macroscopic appearance, the numbers of splenic CD11b^+^Gr-1^+^ cells also were lower in mice treated with Ad-*Tff2* or Ad-*Tff2*-CTP-Flag (Fig. 2c,f,i,k). Moreover, administration of Ad-*Tff2*-CTP-Flag also decreased the numbers of macrophages/granulocytes colony-forming units (cfu) derived from the spleens of these treated mice (Fig. 2j).

**Fig. 3:**
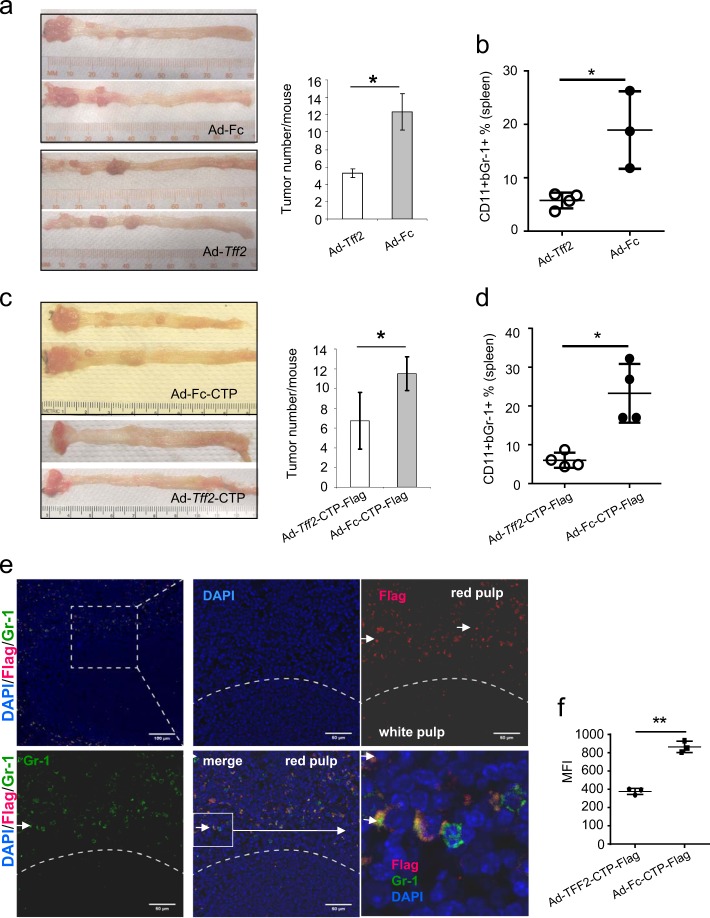
Validation of Ad-*Tff2*-CTP-Flag activity in AOM/DSS-induced colon cancer model. **a**, **b** Ad-*Tff2* suppresses colon tumorigenesis in wild-type mice treated with AOM/DSS. **a** Colon appearance (left), tumor number (right), **b** number of splenic CD11b^+^Gr-1^+^ cells. Two experiments have been done with 3–4 mice in each group. **c**, **d** Ad *Tff2*-CTP-Flag suppresses colon tumorigenesis in AOM/DSS-induced colon cancer model. **c** Colon appearance and tumor number, **d** proportion CD11b^+^Gr-1^+^ cells in the spleen from wild-type mice treated with Ad-*Tff2*-CTP versus Ad-Fc-CTP, unpaired *t*-test, *p < 0.05. Two experiments have been done with 3–4 mice in each group. **e** Overlapping staining for Flag and Gr-1 in the spleen of *Tff2*-null mice injected with Ad-*Tff2*-CTP-Flag. Mice were given 2% DSS for 5 consecutive days, then seven days later adenovirus was administrated via tail vein and mice were sacrificed in one week after adenovirus administration. Bar size is 50 µm. **f** BrdU incorporation in splenic CD11b^+^Gr-1^+^ cells of wild-type mice injected with Ad-Fc-CTP-Flag compare with Ad-*Tff2*-CTP-Flag, unpaired t-test. Wild-type mice were treated 2% DSS for 5 consecutive days, then on day 7 post treatment with DSS single injection of Ad-*Tff2*-CTP or Ad-Fc-CTP-Flag (both 5 × 10^8^ pfu) was administered via tail vein injection. Mice were sacrificed on day 19 after start of DSS treatment, BrdU was injected three hours before sacrifice

To test the potential of adenoviral delivered TFF2-CTP-Flag in cancer therapy, wild-type mice were subjected to AOM/DSS treatment to induce colon carcinogenesis. Six weeks after AOM injection, we administered 5 × 10^8^ pfu per mouse Ad-*Tff2*-CTP-Flag (or Ad-*Tff2*) and Ad-Fc-CTP-Flag (or Ad-Fc) every two weeks, and sacrificed the animals at 16 weeks after the first injection of adenovirus. Given that the development of the colonic tumors in this model requires several months, while adenoviral vector-mediated gene expression is more short-lived, we repeated the adenovirus injections five times (e.g., every few weeks). Similar to mice treated with Ad-*Tff2* versus Ad-Fc (Fig. 3a), animals with administered Ad-*Tff2*-CTP-Flag formed fewer tumors than animals that received the control Ad-Fc-CTP-Flag (Fig. 3c). Consistently, Ad-*Tff2*-CTP-Flag as well Ad-*Tff2* treated animals showed lower proportions of MDSC in their spleens compared to Ad-Fc-CTP-Flag and Ad-Fc control mice (Fig. 3b, d).

Since our previous studies showed that CD11b^+^Gr-1^+^ cells are a target for TFF2 [1], we next investigated whether the localization of TFF2 in the spleen corresponded with myeloid CD11b^+^Gr-1^+^ cells. Mice were treated with DSS, injected with Ad-*Tff2*-CTP-Flag as described above, and the spleen was stained with the antibodies to Flag and Gr-1 (Fig. 3e). These studies revealed that staining for Flag overlapped strongly with the marker Gr-1 in the red pulp of the spleen, the location where myeloid CD11b^+^Gr-1^+^ cells typically reside. Furthermore, in accordance with previous data, we found higher levels of BrdU incorporation in the splenic CD11b^+^Gr-1^+^ cell population in mice injected with Ad-Fc-CTP, in comparison with mice injected with Ad-*Tff2*-CTP-Flag, demonstrating that TFF2-CTP treatment suppresses the proliferation of myeloid cells (Fig. 3f).

### Fusion TFF2-CTP-Flag circulates longer and keeps ability to suppress proliferation of CD11b^+^Gr-1^+^ cells

Finally we compared the duration of residence of wild-type purified recombinant TFF2 protein (Supplementary Fig. 1a) versus recombinant fusion TFF2-CTP-Flag (Supplementary Fig. 1b) in the circulation of *Tff2*-null mice. The fusion recombinant TFF2-CTP-Flag was produced in Freestyle 293-F cells, and displayed in western blot an apparent molecular mass of approximately 38–40 kDa, with the increase in size attributable to hyperglycosylation in Freestyle 293-F cells. Therefore, the comparison between wild-type TFF2 and fusion TFF2-CTP-Flag was done on the base of equimolar concentrations of the injected proteins. This allows for equimolar comparison despite of difference in molecular weight.

**Fig. 4:**
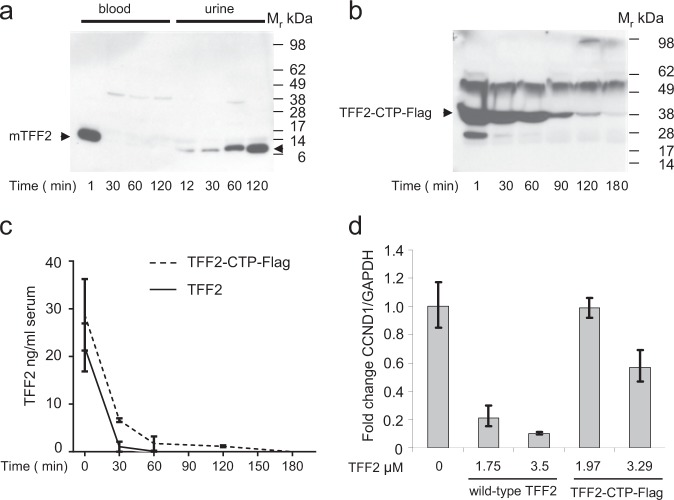
Clearance of recombinant mouse TFF2 and fusion TFF2-CTP-Flag from the blood. **a**–**c** *Tff2-*null mice were tail vein injected with equal molar amount of purified recombinant TFF2 and TFF2-CTP-Flag per kg of mouse weight. At indicated time points blood was taken and assayed for TFF2 and TFF2-CTP-Flag by western blot with antibodies produced against C-terminus of TFF2 molecule (**a**, **b**) and ELISA (**c**). Excretion of TFF2 with the urine was analyzed by western blot (**a**). ELISA data are plotted as a mean of value and standard deviation at each time point. **d** Dose-dependent downregulation of CCND1 mRNA in CD11b^+^Gr-1^+^ cells upon administration of recombinant wild-type TFF2 and fusion TFF2-CTP-Flag protein. *Tff2*-null mice were given 2.5% DSS for 5 days, then CD11b^+^Gr-1^+^ were sorted and cultured with recombinant TFF2 and TFF2-CTP-Flag in indicated concentrations for a 7 days

In accordance with previous reports, the level of injected mouse TFF2 decreased rapidly in the circulation, reaching undetectable levels by 30 min, and instead appearing gradually in the urine as shown by western blot (Fig. 4a). The level of injected fusion TFF2-CTP-Flag from Freestyle 293-Fcells decreased much less quickly, with higher levels evident by western blot at later time points (Fig. 4b). This result was confirmed by ELISA, where we found a decrease over time in the levels of both wild-type TFF2 and the fusion TFF2-CTP-Flag after administration; nevertheless, the levels of TFF2-CTP-Flag remained much higher than that of wild-type TFF2 (Fig. 4c). We tested the bioactivity of fusion TFF2-CTP-Flag in an in vitro assay using CD11b^+^Gr1^+^ cells sorted from the spleen of *Tff2*-null mice with DSS-induced colitis. CD11b^+^Gr1^+^ were cultured 7 days in the presence of wild-type TFF2 and TFF2-CTP-Flag. Then, total mRNA was isolated from these myeloid cells and cell cycle regulator cyclin D1 mRNA was measured. We found that similar to wild-type TFF2 protein, fusion TFF2-CTP-Flag downregulated cyclin D1 mRNA expression in concentration-dependent manner, although not quite to the same extent as wild-type TFF2 used at the equal concentration (Fig. 4d). Thus fusion TFF2-CTP-Flag still retains its activity along with a prolonged circulatory half-life.

## Discussion

TFF2 is attractive candidate for clinical use in cancer therapy, given its ability to suppress MDSCs and enhance CD8+ T cell infiltration of colorectal tumors [1]. However, TFF2 has in the past showed poor pharmacokinetics, with little effort to date to improve its half-life in circulation. In this study, we investigated a potential strategy to prolong the circulation half-life of TFF2 by using an adenovirus construct (Ad-*Tff2*-CTP-Flag) that translates a fusion peptide, TFF2-CTP-Flag, in which a CTP sequence has been added to the C-terminus of TFF2 molecule, under assumption that the C-terminus modification does not significantly affect bioactivity. This assumption arises from data on single-domain trefoil peptides, TFF1 and TFF3 that share 30–40% homology with the two-domain TFF2, and form homo-dimers through conserved unpaired cysteine residues at their C-terminal ends. Homo-dimers are more potent compared to monomer forms in tests for motogenic or mucosal healing activity, suggesting that the C-terminus is not involved directly in bioactivity, such as receptor binding or affinity [13–16]. However, blockade of both N-part and C-part of TFF1 molecule with neutralizing antibodies did decreased the ability of TFF1 to inhibit the growth of kidney stones, and the C-terminus seems particularly important in the inhibition of oxalate crystal growth by TFF1 [17].

Considering the diversity of biological functions of trefoil peptides, the lack of experimental data on active sites, and the absence of structure- and sequence-based approaches to predict structure- function relationships, it has been difficult to devise a potential strategy to improve TFF2 pharmacokinetics. Thus, we genetically fused the C-terminal end of TFF2 with a CTP sequence that has previously been shown to not affect secretion, receptor binding affinity, or bioactivity, but significantly extended half-life of several hormones. However, a reduction of bioactivity after an addition of CTP tag at least one study has reported; for example, the antiviral and anti-proliferative activity of recombinant human INF fused with CTP significantly decreased in vitro bioassay nevertheless, in vivo activity was not blocked or impaired because of extended time in circulation [18]. Test on in vitro activity do not take in account pharmacokinetic differences and tissue distribution therefore decrease in potency seen in vitro may be compensated in vivo with prolonged time of exposure to target [19].

Similar, after modification of the C-terminus with CTP, TFF2 still retained the ability to decrease tumor growth via suppression of myeloid cells in vivo. Level of both wild-type TFF2 and fusion TFF2-CTP-Flag decreased rapidly after tail vein injection; nevertheless, TFF2-CTP-Flag could still be detected at later time points. Thus, while further modifications may be needed, our study provides a solid basis to develop therapeutic TFF2 peptides to improve its pharmacokinetic and thus to bring new myeloid targeted therapies to the bedside.

## Electronic supplementary material

Supplemental Figure 1 Legend

Supplemental Figure 1
