# Supplementary material for: Therapeutic potential of adenovirus-mediated TFF2-CTP-Flag peptide for treatment of colorectal cancer
Source: Cancer Gene Ther. 2018 Jul 25;26(1):48–57. doi: 10.1038/s41417-018-0036-z (PMC6760534; doi:10.1038/s41417-018-0036-z)
Supplement: Supplementary file 1 — Supplemental Figure 1 Legend [file 41417_2018_36_MOESM1_ESM.doc]

Supplementary figures 1. Purity of recombinant wild-type TFF2 (>95%) and fusion TFF2-CTP-Flag (around 95%) shown in SDS-PAGE stained with Coomassie Blue R250. Purified TFF2 under non-reducing and reducing conditions (a) and TFF2-CTP-Flag under reducing conditions (b).
