# Supplementary figures and images for: Therapeutic potential of adenovirus-mediated TFF2-CTP-Flag peptide for treatment of colorectal cancer
Source: Cancer Gene Ther. 2018 Jul 25;26(1):48–57. doi: 10.1038/s41417-018-0036-z (PMC6760534; doi:10.1038/s41417-018-0036-z)

**a**

$M_r$  kDa

148  
98  
64  
50  
36  
22  
16  
9  
6  
4

TFF2 ►

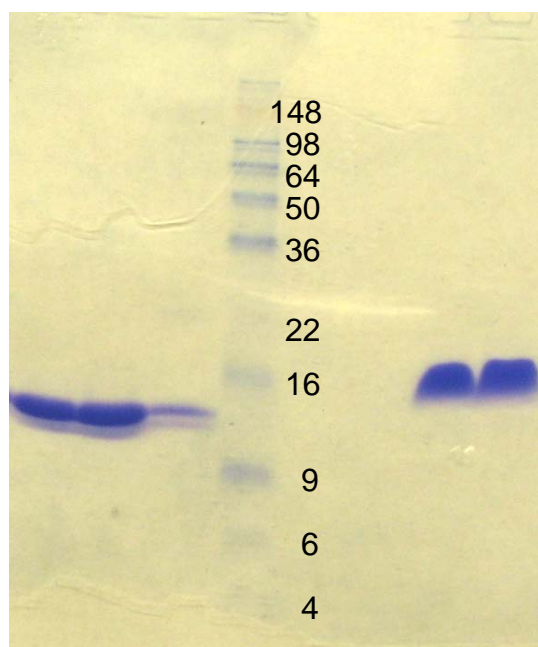

Non-reduced

reduced

**b**

$M_r$  kDa

80  
60  
50  
40  
30  
25

TFF2-CTP-Flag ►

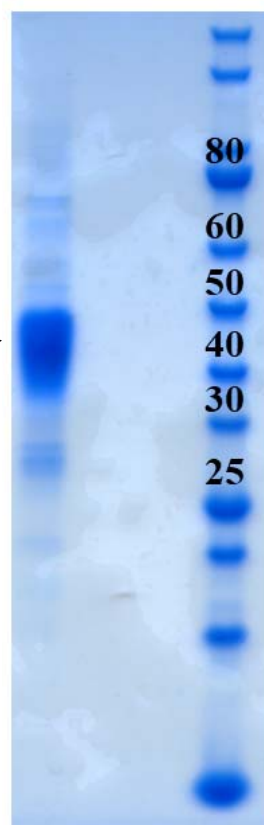

reduced

Supplement: Supplementary file 2 — Supplemental Figure 1 [file 41417_2018_36_MOESM2_ESM.pdf]
